# CAraCAl: CAMML with the integration of chromatin accessibility

**DOI:** 10.1186/s12859-024-05833-3

**Published:** 2024-06-13

**Authors:** Courtney Schiebout, H. Robert Frost

**Affiliations:** Department of Biomedical Data Science, Dartmouth College, Hanover, NH 03766 USA

**Keywords:** scATAC-seq, scRNA-seq, Gene activity, Cell typing

## Abstract

**Background:**

A vital step in analyzing single-cell data is ascertaining which cell types are present in a dataset, and at what abundance. In many diseases, the proportions of varying cell types can have important implications for health and prognosis. Most approaches for cell type annotation have centered around cell typing for single-cell RNA-sequencing (scRNA-seq) and have had promising success. However, reliable methods are lacking for many other single-cell modalities such as single-cell sequencing assay for transposase-accessible chromatin (scATAC-seq), which quantifies the extent to which genes of interest in each cell are epigenetically “open” for expression.

**Results:**

To leverage the informative potential of scATAC-seq data, we developed CAMML with the integration of chromatin accessibility (CAraCAl), a bioinformatic method that performs cell typing on scATAC-seq data. CAraCAl performs cell typing by scoring each cell for its enrichment of cell type-specific gene sets. These gene sets are composed of the most upregulated or downregulated genes present in each cell type according to projected gene activity.

**Conclusions:**

We found that CAraCAl does not improve performance beyond CAMML when scRNA-seq is present, but if only scATAC-seq is available, CAraCAl performs cell typing relatively successfully. As such, we also discuss best practices for cell typing and the strengths and weaknesses of various cell annotation options.

## Introduction

Single-cell technologies have grown massively in the past decade, becoming faster, more precise, and more accessible [1–5]. This has allowed for utilization of single-cell modalities, in particular single-cell RNA-sequencing (scRNA-seq), across many organisms, tissues, and diseases; however, many challenges remain with leveraging these data sources. Single-cell methods are often sparser and noisier than their bulk counterparts and their dissociation protocols do not maintain tissue architecture or organization, making cell typing a challenge [6, 7]. More recent technologies are finding paths around this, particularly in spatial transcriptomics where RNA can be quantified without altering tissue architecture, but the challenge of annotating the cell identities of existing datasets (and future datasets that do not leverage these newer methods) remains [8–11].

Being arguably the most prevalent and accessible single-cell modality, many cell typing tools for scRNA-seq have been developed to address the annotation challenges for this type of data. These methods typically address the challenge of sparsity in one of two ways: either by aggregating cells for higher-powered categorization (cluster-based methods) [12–15] or by categorizing cells based on a group of markers or expression correlation [16–19]. Both types of methods have found relative success, although the latter is generally considered more statistically sound as it prevents “double-dipping” in assuming that the clusters used for cell aggregation were correctly delineated since each cell is treated individually [20].

While these methods have certainly eased the challenge of cell typing in scRNA-seq, there are many other single-cell omics modalities that have much less developed cell typing methodologies. One such modality is single-cell epigenomics. In the past decade, there have been several developed methods to address quantifying single-cell epigenomes [21]. Methods for single-cell bisulfite sequencing and single-cell chromatin immunoprecipitation (ChIP) have been developed [21–23]. However, these methods struggle from methodological or output complications, like difficult protocols or excessively sparse results, that have prevented broad uptake [21–23]. By far the most commonly used single-cell epigenomics method thus far is single-cell sequencing assay for transposase-accessible chromatin (scATAC-seq) [24, 25]. This technology allows for the quantification of all locations, or peaks, in a genome where the chromatin is accessible [24, 25]. These peaks can then be annotated for the gene near which they are located, giving a quantification for which genes in each cell are capable of being expressed, even if they are not actively being expressed at the time [26, 27]. Given that epigenetic alterations in part allow for the specialization of cell types, utilizing this information, either with scRNA-seq or independently, has great potential for cell type annotation [28, 29]. Indeed, previous work in bulk deconvolution has shown that methylation-based epigenetic profiles can recapitulate cell populations with high accuracy [30, 31]. While not the same as scATAC-seq, this is an encouraging finding that epigenetics can be informative for cell identification.

Some methods have been developed to carry out cell typing of scATAC-seq data, but these have almost entirely been realized using scRNA-seq as a reference or to work in tandem with scRNA-seq data, such as scJoint [32] and Seurat transfer learning [27, 33]. Very few methods have been developed using scATAC-seq as both the reference and the test data. While there are certainly advantages in combining scRNA-seq and scATAC-seq for cell typing, the resulting interdependence limits the applicability of these methods to cases where scRNA-seq is not present or highly reliable. Furthermore, by utilizing scRNA-seq as a reference, any features unique to scATAC-seq data may be lost.

To navigate these challenges, we developed CAMML with the integration of chromatin accessibility (CAraCAl). CAraCAl leverages the framework of our previous method for scRNA-seq, CAMML [19], but with gene sets and a scoring framework customized specifically for the unique features of scATAC-seq data. These customizations allow CAraCAl to be biologically interpretable, rather than a black box for cell type annotation that cannot be verified. In addition, by utilizing gene sets based on scATAC-seq, CAraCAl enables users to perform cell type analysis designed for scATAC-seq, rather than using the other common methods currently existing in the space that use scRNA-seq as a reference. We demonstrate CAraCAl’s novel utility by evaluating how scATAC-seq differs from its scRNA-seq counterpart, even in the same dataset. We then test how cell typing improves in scATAC-seq datasets when gene sets are built specifically for scATAC-seq data characteristics, using both manually annotated and sorted scATAC-seq data for benchmarking. Lastly, we discuss the relative strengths and weaknesses of the various modalities that can be used for cell typing, and how they might be best leveraged depending on the needs of a particular investigation.

## Methods

### Data processing

Three publicly available datasets were used to evaluate the performance of CAraCAl: joint scRNA-seq/scATAC-seq peripheral blood mononuclear cell (PBMC) data from 10X Genomics [34], scATAC-seq data from bone marrow and PBMCs that was manually annotated for cell identity [35], and scATAC-seq data for sorted PBMC immune cell types [36]. The first of these datasets allows for comparison between the performance of scATAC-seq cell typing and scRNA-seq cell typing, while the latter two give semi-gold standard cell type annotations with which accuracy calculations can be performed.

#### Joint scRNA-seq/scATAC-seq dataset

The joint scRNA-seq/scATAC-seq PBMC data from 10X Genomics was processed and analyzed using Seurat (v5.0.1) and Signac (v1.12.9) [27, 33]. The chromatin assay was built with genome annotations from Homo sapiens Ensembl database v86 [37]. Cells with fewer than 1000 counts for scRNA-seq or scATAC-seq and cells with greater than 25,000 or 100,000 counts for scRNA-seq and scATAC-seq respectively were excluded from further analysis. This resulted in a dataset containing 36,601 genes and 111,978 peaks across 9229 cells. SCTransform was performed on the scRNA-seq data across 3000 variable features to normalize and scale the data [38]. Signac’s GeneActivity function was applied to the peak data to estimate gene expression based on chromatin accessibility [27]. This estimated gene activity was then treated as RNA data and normalized and scaled with SCTransform on the 3000 most variable features [38]. Both the original RNA data and the estimated gene activity were then visualized using Uniform Manifold Approximation Projection (UMAP) on 30 principal components (Fig. 2A) [33, 39].

To set a standard for comparing the performance of both scRNA-seq and scATAC-seq cell typing methods, Seurat label transfer was used to annotate the joint dataset [33, 40]. The joint data was mapped onto the multimodal reference dataset available in the 2021 paper from Hao, et al., which contains labeled cells for all of the major immune cells types [40]. However, this approach for cell annotation does have some limitations. First, this method for categorization is not an objective ground truth, so some bias may be introduced from the reference data, and second, this method may be biased towards the performance of the scRNA-seq cell typing because that is the modality the transfer anchors are built upon. While not perfect, having some reference for the overall performance of each modality allows for more comparable evaluations.

#### Manually annotated dataset

The Satpathy manually annotated dataset was made available by the authors includes gene expression estimates generated using Cicero [26, 35]. The data was transformed to raw count form for analysis and progenitors and basal cells were removed in order to be consistent with the other datasets. This estimated RNA data was then log normalized and centered and scaled across 2000 variable features with Seurat [33]. The traditional Seurat “log-normalization” pipeline was used in lieu of SCTransform for this dataset. This was primarily opted for because this data was much larger and was already annotated, thus it did not require SCTransform for label transfer. The estimated gene activity data was then visualized using Uniform Manifold Approximation Projection (UMAP) on 30 principal components (Fig. 4A) [33, 39].

Upon discovery that gene sets built with Signac gene activity do not perform well in detecting cell identities in datasets built from Cicero, a second Cicero-based manually annotated dataset from Granja, et al. was accessed [41]. This dataset was processed similarly: PBMCs identified as B cells, monocytes, NK cells, or T cells were kept for downstream analysis. These cells were then log-normalized and scaled and centered across 2,000 variable features. The resulting data was then visualized using Uniform Manifold Approximation Projection (UMAP) on 30 principal components (Fig. 4B) [33, 39].

#### Sorted dataset

The scATAC-seq data from the sorted data was available for each cell separately, so each dataset was processed as peak data and mapped to gene activity individually [36]. The data contained datasets for the following immune cell types: monocytes, B cells, NK cells, and CD4 and CD8 T cells [36]. Each dataset was processed with genome annotations from Homo sapiens Ensembl database v86 [37] and only cells with at least 200 features were kept for downstream analysis [33]. The resulting datasets contained the following cell counts: 2436 monocytes, 4536 B cells, 3981 NK cells, and 5310 CD4 and 4999 CD8 T cells. Once each dataset’s gene activity was calculated, the assays were merged into one. Once merged, only genes present in at least 100 cells and cells with at least 100 genes were kept. This merged data, with 14,013 features for 19,593 cells, was then log-normalized and scaled and centered across 2000 variable features with Seurat [33]. The merged estimated gene activity data was then visualized using Uniform Manifold Approximation Projection (UMAP) on 30 principal components (Fig. A) [33, 39].

### CAraCAl method

#### VAM and CAMML

The CAMML (Cell typing using variance Adjusted Mahalanobis distances with Multi-Labeling) method was developed as a cell typing technique for scRNA-seq data that leverages the single-cell gene set enrichment analysis method Variance Adjusted Mahalanobis (VAM) [19, 42]. In short, the method works by taking gene sets that represent up-regulated genes in a cell type of interest and scoring each cell for those genes using a version of the Mahalanobis distance that is optimized for the characteristics of single-cell gene expression data [19, 42]. These VAM distances can then be mapped to a gamma distribution in order to convert each distance into a score according to the null cumulative distribution function (CDF) [42]. This allows each gene set in each cell to be scored from 0 to 1, enabling both statistical inference (i.e., the CDF values can be converted into p-values) and comparison of scores for different gene sets on the same scale, regardless of gene set size [42].

CAMML expanded upon this framework by developing gene sets specific to cell types, with a particular emphasis on immune cell types, and by building out options for labeling based on the cell type scores [19]. This allowed each individual cell in a scRNA-seq dataset to be either single- or multi-labelled as cell types of interest and enabled sensitive and accurate labeling of immune cells in peripheral blood mononuclear cells (PBMCs) and tumor microenvironments (TMEs), in both humans and mice [19]).

#### Adapting CAMML for down-regulation

VAM and, given its dependency, CAMML previously only utilized up-regulated genes in their gene sets as the distance calculation accounted for the extent to which gene expression exceeded zero [19, 42]. However, given that an absence of gene expression is often as indicative of cell identity as high expression, developing a method for including down-regulated genes in the scoring of cell types was a priority. To address this, we updated CAMML to allow the inclusion of two gene sets for each cell type, one containing genes that are expected to be up-regulated and one containing genes that are expected to be down-regulated [43]. Each of these gene sets is then scored by VAM individually [42]. These scores are then combined for each cell type in each cell as the sum of the up-regulated VAM score and the inverse of the down-regulated VAM score (Eq. 1) to result in a single score for each cell [43]. This score then represents the combined strength of a cell’s up- and down-regulated genes for a cell type.

1 $$\begin{aligned} VAM_{i,j} = VAM(up)_{i,j}+(1-VAM(down)_{i,j}) \end{aligned}$$

### Gene set development

**Fig. 1:**
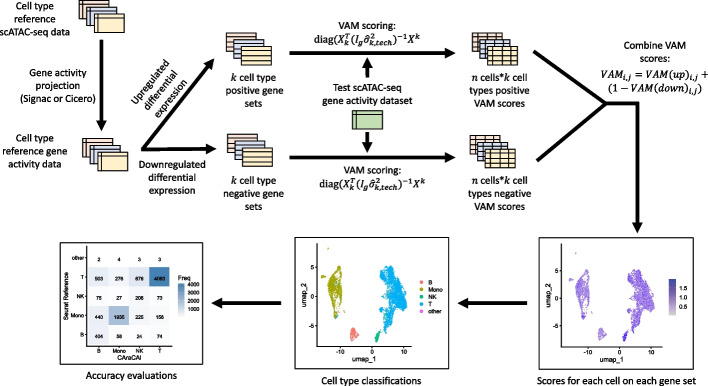
Methodological workflow of CAraCAl cell typing

Upon testing gene activity estimated from scATAC-seq data on pre-made CAMML gene sets built for scRNA-seq, it became clear that these gene sets did not translate well to the new modality [19]. Thus, new gene sets were developed based on the differential expression of the estimated gene activity of the cell types provided in either the Granja manually annotated dataset or the sorted dataset, depending on whether Cicero or Signac were used for expression estimation [26, 27, 36, 41]. For each cell, two gene sets were developed in accordance with the up- and down-regulation integration outlined in Eq. 1: one containing the most up-regulated genes in each cell type versus all others and one containing the most down-regulated genes in each cell type versus all others. This differential expression was calculated based on a Wilcoxon Rank Sum test in Seurat [33, 44]. For each gene set, the top (and bottom) 100 genes by log2 fold-change were selected. This value was intentionally selected to be higher than the number of genes required for scRNA-seq in order to bypass the challenges of noise and sparsity the projected gene activity matrices presented, beyond those typically seen in scRNA-seq. Each gene in each gene set was then weighted by the fold-change of that gene’s activity in differential expression analysis. A workflow of the CAraCAl process is outlined in Fig. 1.

### Performance evaluation

#### CAMML

Upon initial evaluation of gene activity estimated from scATAC-seq data, it was hypothesized that the gene sets already built in CAMML for scRNA-seq may also serve as useful cell type gene sets for scATAC-seq. CAMML was thus performed on both the scRNA-seq data and scATAC-seq based gene expression estimates for the joint scRNA-seq/scATAC-seq dataset [19, 34]. The scATAC-seq CAMML scores were then evaluated for agreement with the scRNA-seq CAMML annotations and with the Seurat label transfer annotations [19, 40]. Following this, the scATAC-seq CAMML scores were also integrated into the scRNA-seq CAMML scores to glean whether they contributed to increased accuracy when compared with the Seurat label transfer annotations [19, 40]. The integrated score was defined as the product of the scRNA-seq CAMML score and the binary of the scATAC-seq CAMML score: 0 if the score was less than the median for all scores of that cell type and 1 if the score was greater than the median for all scores of that cell type. This is akin to one of the methods utilized for Cellular Indexing of Transcriptomes and Epitopes by sequencing (CITE-seq) in CAMML with the Integration of Marker Proteins (ChIMP) [45] and is visualized in Eq. 2.

2 $$\begin{aligned} Integrated CAMML_{i,j}&= CAMML(RNA)_{i,j} * \nonumber \\ (CAMML(ATAC)_{i,j}&> Med(CAMML(ATAC)_j)) \end{aligned}$$

#### CAraCAl

To evaluate the performance of CAraCAl, several tests were performed. The gene sets built with the sorted data were used to score and identify cell types in the joint scRNA-seq/scATAC-seq data and compared for agreement with the assignments found using CAMML and with the annotations assigned by Seurat label transfer [19, 34, 36, 40]. The sorted data gene sets were also used to evaluate the performance of CAraCAl on the Satpathy manually annotated dataset for accuracy [35, 36]. To better account for the different gene activity projection methods, gene sets built from the Granja manually annotated dataset were also tested on the Satpathy dataset and evaluated for accuracy [35, 41]. Lastly, both gene sets built were also used to evaluate the performance of CAraCAl on the datasets they were built from [36, 41]. While inherently biased, ensuring that the gene sets indeed found differential signals in their source datasets was deemed worth investigating.

In addition to comparison with cell type annotations from manual classification and sorting, we evaluated time efficiency on the Granja dataset, both in its entirety and on subsets of the data [41]. The Granja dataset was selected because it is the largest, allowing for thorough evaluation of timing across gene and cell numbers [41]. We compared CAraCAl’s timing with the processing time of Seurat label transfer, although of note, given that this is a method for labeling with RNA, the methodologies are not performing the same analysis [33, 40]. Linear regression was performed to evaluate how each method’s time requirement was altered by increases in the number of genes and cells provided in the dataset, as well as the interaction between them.

## Results and discussion

### Joint scRNA-seq/scATAC-seq dataset

#### Testing with CAMML

**Fig. 2:**
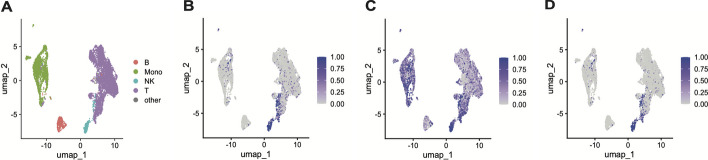
CAMML scores for the built-in NK cell gene set on the joint scRNA-seq and scATAC-seq PBMC data from 10X Genomics. **A** UMAP of the joint scRNA-seq/scATAC-seq data, colored by identity. **B** The CAMML scores for NK cells on the scRNA-seq assay from this dataset. **C** The CAMML scores for NK cells on the gene activity estimates generated from the scATAC-seq data. **D** The integrated CAMML scores generated on scRNA-seq and scATAC-seq data [34]. For clarity of the comparison, the UMAP computed on the scATAC-seq based gene activity estimates is used to illustrate the scores in all three plots

**Fig. 3:**
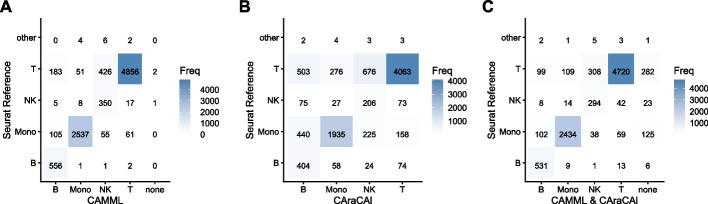
Confusion matrices of CAMML, CAraCAl, and integrated CAMML and CAraCAl annotations versus those identified by Seurat in the joint scRNA-seq/scATAC-seq dataset. **A** CAMML identified cell types by scRNA-seq, **B** CAraCAl identified cell types by scATAC-seq, and **C** integrated CAMML and CAraCAl identified cell types versus those identified by Seurat label transfer [34, 40]

Given that scATAC-seq gene activity estimates are intended to capture similar information as scRNA-seq, it was hypothesized that scATAC-seq gene activity estimates could be successfully used with the existing CAMML pipeline to annotate cell types [19]. To test this, the built-in immune cell gene sets included with the CAMML R package were applied using the CAMML technique to both the scRNA-seq and scATAC-seq in the joint dataset to compare their classification performance [34]. Both sets of CAMML results were also compared to the results of Seurat label transfer to give an unrelated reference source [40]. As illustrated in Fig. 2, CAMML applied to the scRNA-seq data provided more specific scoring, in this case of NK cells (Figs. 2A and 3A) as compared to CAMML applied to the scATAC-seq data, which generated relatively more noisy scores that prevented a strong visual discretization of NK cells (Fig. 2B).

To evaluate if cell typing using both scATAC-seq and scRNA-seq data would have better accuracy than scRNA-seq alone, the CAMML outputs generated using the two data types were integrated via the method outlined in Equation 2. Here, we found that the inclusion of scATAC-seq cell typing results did not contribute to the visual discretization of NK cells (Fig. 2C). Further, when evaluating each of the three CAMML methods (CAMML on scRNA-seq, CAMML on gene activity of scATAC-seq, and the integration of both prior methods) against Seurat label transfer, we did not find that scATAC-seq CAMML was highly accurate on its own nor did it contribute to any improvement when integrated with the scRNA-seq CAMML. When CAMML was executed on just the scRNA-seq data, CAMML identified the same cell type as Seurat label transfer 90% of the time. By contrast, when CAMML was executed on the scATAC-seq data, this value was only 71%. When CAMML results for the two data types were integrated, the agreement with Seurat label transfer was only 74%, indicating that the scATAC-seq did not aid in improving the performance up to or beyond what scRNA-seq CAMML could do alone.

Given this finding, we determined that developing a cell typing method specifically designed for scATAC-seq datasets was necessary for successful annotation. Despite their shared data structure, the gene activity estimates generated from scATAC-seq data were not reflective of the actual pattern of gene expression in those cells. Thus, we developed and tested this and additional datasets using gene sets built for scATAC-seq.

#### Testing with CAraCAl

To evaluate cell typing performance using both up- and down-regulated gene sets built from scATAC-seq gene activity data, we applied the CAraCAl method to the joint dataset and compared the results to both the standard CAMML method (i.e, CAMML executed on the scRNA-seq data) and Seurat label transfer annotations [34, 40]. The cell type annotations from CAraCAl had a 68% agreement with the CAMML annotations and a 71% agreement with Seurat label transfer (Fig. 3B). However, upon integrating these scores with the scRNA-seq CAMML scores (as in Eq. 2), the resulting agreement with Seurat label transfer increased to 85% (Fig. 3C), indicating that, despite its similar accuracy to the RNA-based gene sets, these updated gene sets appear to contribute more useful information regarding cell identities. While this combination still does not surpass the performance of CAMML using just RNA-based gene sets, the improved accuracy of the combined method indicates the benefit of using scATAC-seq specific gene sets for the analysis of scATAC-seq data. In addition, given that the reference annotations for this dataset were based on scRNA-seq data, it is not necessarily surprising or discouraging that the RNA-based method outperforms any scATAC-seq method.

### Manually annotated datasets

#### Testing the Satpathy dataset

The Satpathy manually annotated dataset was analyzed in the same way as the joint scRNA-seq/scATAC-seq dataset as outlined in Sect. 3.1.2 above, with up- and down-regulated gene sets built from the differential gene expression between different cell types in the sorted data [35, 36]. The top scoring cell type for each cell was then compared to the manual annotations to ascertain accuracy. In contrast to the evaluation of the joint scRNA-seq/scATAC-seq dataset, the success rate for CAraCAl was much lower: only about 48% of cells were correctly categorized. We hypothesized that was due to the fact that the scATAC-seq data made available from this study was gene activity estimation performed using Cicero rather than Signac [26, 27].

**Fig. 4:**
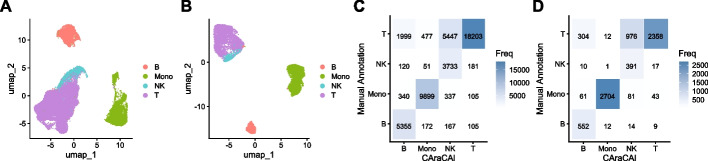
Confusion matrices of CAraCAl cell types versus manual annotation on the Satpathy and Granja datasets. **A** UMAP of the Satpathy dataset, colored by manual annotation. **B** UMAP of the Granja dataset, colored by manual annotation. **C** CAraCAl cell type annotations versus manual annotations for the Satpathy dataset, using gene sets built from the Granja dataset **D** CAraCAl cell type annotations versus manual annotations for the Granja dataset, using gene sets built from the Granja dataset [35, 41]

To test this, gene sets were instead built using the manually annotated dataset from Granja, et al., which also employed Cicero for estimating gene activity [41]. When the Satpathy manually annotated data was cell typed based on these gene sets, the accuracy was much higher, at 81% (Fig. 4A), indicating that there was indeed a limitation to applying gene activity estimated using Signac to datasets built with Cicero.

#### Testing the Granja dataset

For additional validation, the Granja-based gene sets were also tested on the Granja manually annotated data [41]. While this is inherently biased, validation on as many datasets as possible, given the limited number of annotated scATAC-seq datasets, was deemed worth pursuing. The gene sets predicted the same cell type as the manual annotation with 80% accuracy (Fig. 4B), indicating that the gene sets did, for the most part, successfully detect the genes necessary for differentiating cell types within the data.

**Fig. 5:**
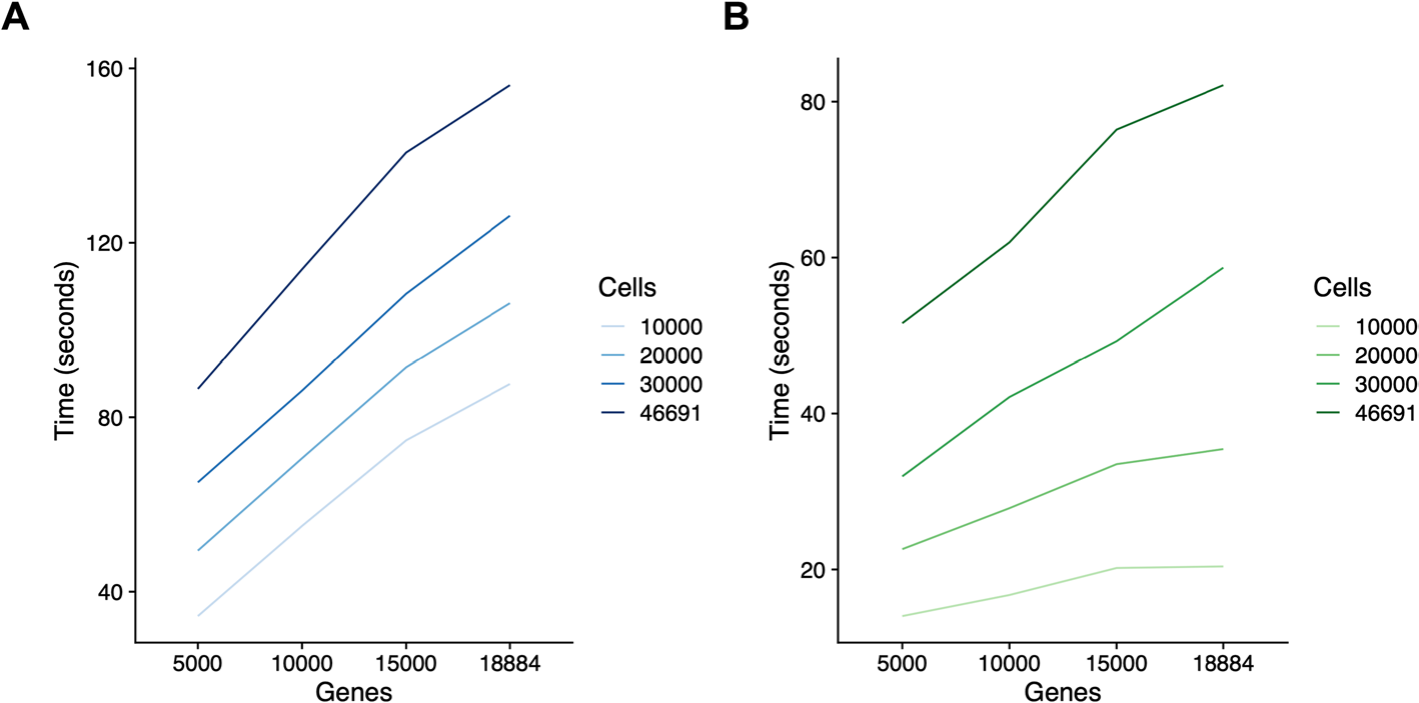
Timing of subsets of the Granja dataset using CAraCAl and Seurat label transfer. **A** CAraCAl timing and **B** Seurat label transfer timing

Time efficiency was evaluated on the Granja dataset for CAraCAl and Seurat label transfer [33, 40]. Timing was evaluated on each combination of 5000, 10,000, 15,000, and the maximum 18,884 genes, as well as 10,000, 20,000, 30,000, and 46,691 cells. Generally, CAraCAl takes about twice as long as Seurat label transfer, but both run quite efficiently (Fig. 5) [33, 40]. In a linear regression analysis of the time by gene and cell numbers and their interaction, CAraCAl timing was significantly increased by genes, cells, and their interaction, while Seurat label transfer timing was significantly increased by cells and the interaction of cell and gene numbers [33, 40]. Of note, the coefficient of the interaction between gene and cell numbers was higher in Seurat label transfer than in CAraCAl, which is visible in the increase in slopes in Fig. 5B. One limitation of this analysis is that Seurat label transfer performs cell annotation based on RNA, rather than ATAC data, preventing an exact comparison of the two methods; however, it was encouraging to find that even in the largest version of the dataset, CAraCAl is carried out in just over 2 minutes [33, 40].

### Sorted dataset

**Fig. 6:**
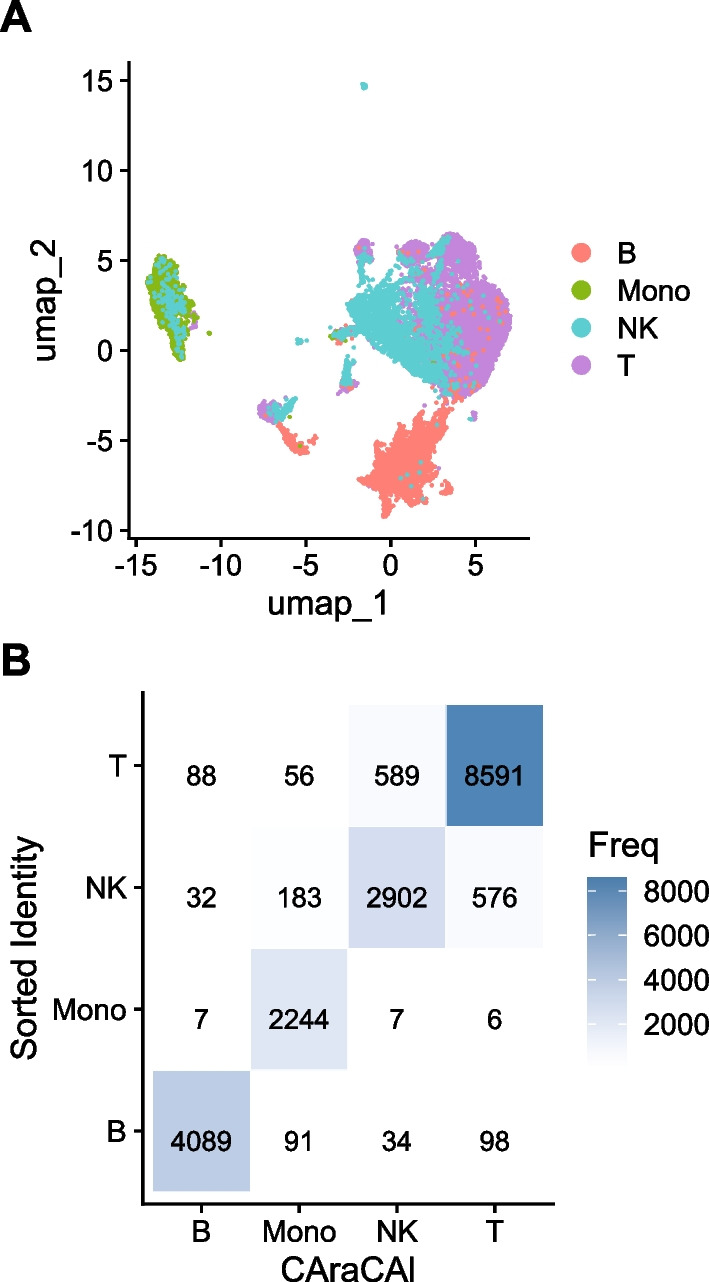
Confusion matrices of CAraCAl cell types versus sorted cell type identities on the Lareau dataset. **A** UMAP of the sorted dataset, colored by the sorted identity. **B** Confusion matrix of the highest scoring cell type for each cell according to CAraCAl and the true identity of that cell according to sorting [36]

While inherently biased, the sorted dataset was tested using the gene sets estimated on the sorted dataset [36]. This was done despite the bias to ensure that the gene sets were indeed capturing gene activity differences between cell types and to glean performance on a gold-standard dataset, and to provide further validation given the minimal number of available annotated scATAC-seq datasets [36]. As expected, CAraCAl performed very well on this data, accurately capturing the correct cell type 91% of the time (Fig. 6). This adds confidence to the prior results of cell typing on datasets without gold standard annotations.

### Cell typing recommendations

**Fig. 7:**
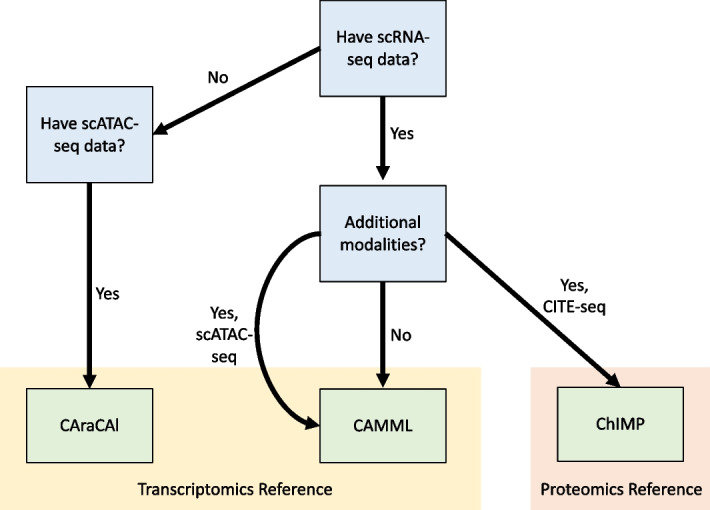
Decision-making workflow for cell typing based on available omics modalities. Blue boxes reflect available test data options, green boxes are methods. The yellow outline highlights methods that use transcriptomics as a gold standard for cell identification. The orange outline reflect the method that uses surface markers for cell identification

Based on the findings in this work and our previous work [19, 45], we created a general set of guidelines for cell typing of single cell data. Of course, the goal of any study will determine the types of single-cell data needed, but, if the overall goal is cell type annotation, a few general recommendations can be made. First, given its strong association with the abundance of the cell surface markers used to define canonical cell types, scRNA-seq data, if available, should always factor into the cell type annotation process. When scRNA-seq is the only data modality available, CAMML has proven to be accurate and reliable, with the added benefit of being customizable to user needs [19]. If the scRNA-seq has been performed in parallel with CITE-seq, the CITE-seq data can contribute to the confidence and specificity of cell type annotations. Thus, utilizing ChIMP in these cases is advised to further validate the cell type assignments [45]. In cases where both scRNA-seq and scATAC-seq are performed, however, CAMML is still the most reliable avenue for cell typing, so it is recommended that the scATAC-seq data is not integrated into the cell typing in this case. In cases where there is only scATAC-seq, CAraCAl can be used to annotate the cell types present, with the same methodological benefits as CAMML, although at generally lower accuracy, as outlined in this work. Furthermore, while the analysis was performed in immune cells to test its utility in this work, given the customizability of CAMML, ChIMP, and CAraCAl, any cell types with available reference data can be annotated. In this vein, there is additionally consideration for what the cell type annotation is based on when choosing a method. CAMML and CAraCAl are built to reflect the cell types identified by transcriptional profiles, whereas ChIMP is built to identify cell types based on their surface protein markers [19, 45]. This decision-making process is outlined in Fig. 7 for simplicity and visualization.

There are, of course, many instances where scATAC-seq is highly useful beyond cell typing, such as when evaluating the accessibility of transcription factors and other regulatory regions. In the case of cell typing, however, scRNA-seq is a far more reliable data type. The reasons for superior performance of cell typing methods that use scRNA-seq data versus cell typing based on scATAC-seq data include:

- Cell identities are often defined by their transcriptional profile, so the modality that utilizes the same data is likely to best recapitulate that profile.
- Chromatin accessibility does not always imply active expression of the associated gene.
- scATAC-seq cell typing is based on projected gene expression, making it a more noisy version of scRNA-seq and requires the assumption that gene mapping is correct.

There are many uses for scATAC-seq, but scRNA-seq appears to be the stronger modality for the goal of cell type annotation.

This is not to say that CAraCAl does not have utility and novelty outside of CAMML. CAraCAl builds gene sets for both down- and upregulated genes based on scATAC-seq gene activity rather than based on reference RNA datasets, allowing for a more scATAC-seq-specific annotation. This combination of considering genes in both directions and building gene sets that are customized to scATAC-seq, both in content and size, allows for a customized user experience with CAraCAl for scATAC-seq specific annotation that is further bolstered by its biological interpretability.

### Limitations

There are some limitations to consider and acknowledge in this work. Overall, the method is weakened by the reliance on gene activity projections, which do not completely recapture the true expression data in cells. This leads to lower accuracy and less confidence in classifications. This is further contributed to by the lack of consistency across gene activity mapping methods, necessitating different gene sets for each. Despite these challenges, there is certainly still utility for this method in cases when only scATAC-seq is available.

The method itself also has a few inherent limitations. CAraCAl relies on gene sets that have to be defined by the user. This prevents CAraCAl from identifying cell types that are not included in the analysis. Thus, in cases where novel or unexpected cell types are of interest, CAraCAl is not well suited to the analysis. Furthermore, given that CAraCAl relies on gene activity quantification, peaks in the scATAC-seq data that are not within or closely up- or downstream from a gene will not be considered. This may prevent cell type-relevant peaks that are distant from a gene from contributing to the quantification of gene activity, and thus may exclude relevant peaks from informing classification overall.

CAraCAl may also be limited by the quality of the input, both the reference and query data. If the reference data is inaccurately annotated, the resulting gene sets may not be a valid reflection of the up- and downregulated genes within that cell type. If the query data is overly noisy or overly sparse, there may be issues with misclassification. Specifically, if the gene activity data is overly noisy, there may be false positive and negative classifications, and if the gene activity data is overly sparse, the method may not have enough information to make valid classifications, resulting in a high false negative rate.

### Future directions

There are many future directions that can be pursued to further strengthen CAraCAl. As more annotated datasets are made available, building out further cell type gene sets for scATAC-seq data could improve the reliability and scope of CAraCAl. Furthermore, integrating additional single-cell epigenetics methods, such as CpG methylation, as they become more prominent could be beneficial in improving the specificity of CAraCAl and other cell typing methods.

In addition, there are many future use cases for CAraCAl in more applied settings. The utility of a biologically transparent method for cell type annotation in scATAC-seq could benefit future research that aims to understand how cell abundances differ between diseased and healthy tissues or over time.

## Conclusion

As the utilization of single-cell methods continues to rise, annotating the cells present when sorting is not available is increasingly necessary; however, this is especially a challenge given the nature of single-cell data, which is often sparse or noisy, preventing single markers from being useful for categorization [6, 7]. Instead, cell annotation requires methods that utilize multiple data points, either by grouping the cells or by considering many genes in classification. Many methods have successfully been developed for scRNA-seq that follow one of these two aggregation approaches, but there is still a need for reliable cell typing techniques for non-transcriptomic single-cell modalities such as scATAC-seq. To address this challenge, we have developed CAraCAl (CAMML with the integration of chromatin accessibility), a cell typing method for scATAC-seq data. This method follows a similar approach as our previous CAMML method for cell typing of scRNA-seq data with two important changes: (1) the cell type gene sets are estimated using gene activity estimated from scATAC-seq data, and (2) support is included for both up-regulated and down-regulated genes in the scoring calculation [19].

Evaluation of CAraCAl on scATAC-seq datasets with gene activity estimated using either Signac or Cicero revealed that the true cell type (as determined by label transfer, manual annotation, or sorting) was correctly identified 70–90% of the time. While this classification performance is lower than what is typical for cell typing of scRNA-seq data, in cases where only scATAC-seq is present, CAraCAl is the best option for gleaning the likely cell type in the often noisy scATAC-seq gene activity data. Furthermore, by building gene sets based on differential expression of scATAC-seq gene activity, CAraCAl ensures transparency and allows for customization, i.e., users can see what genes are important for determining cell identity and can alter the gene set for a specific cell type to best match their experimental goals and data characteristics.

Overall, in cases where only scATAC-seq data is available, CAraCAl serves as a useful cell typing tool. In cases where both scRNA-seq and scATAC-seq are available on the same cells, cell type categorization may be more reliable by leveraging a scRNA-seq cell typing tool, like CAMML. This is especially true given the inconsistency of results between the existing gene activity estimation methods on scATAC-seq, requiring consideration that is not necessary in scRNA-seq cell typing.

## Data Availability

All data used in this manuscript are publicly available at the cited sources. The joint scRNA-seq/scATAC-seq data from 10X is available at: https://www.10xgenomics.com/datasets/pbmc-from-a-healthy-donor-no-cell-sorting-10-k-1-standard-2-0-0. The Satpathy data is available at: https://www.ncbi.nlm.nih.gov/geo/query/acc.cgi?acc=GSE129785. The Lareau data is available at: https://www.ncbi.nlm.nih.gov/geo/query/acc.cgi?acc=GSE123581. The Granja data is available at: https://www.ncbi.nlm.nih.gov/geo/query/acc.cgi?acc=GSE139369.

## Abbreviations

scRNA-seq: Single-cell RNA-sequencing
scATAC-seq: Single-cell sequencing assay for transposase-accessible chromatin
CAraCAl: CAMML with the integration of chromatin accessibility
ChIP: Chromatin immunoprecipitation
PBMCs: Peripheral blood mononuclear cells
UMAP: Uniform manifold approximation projection
CAMML: Cell typing using variance adjusted Mahalanobis distances with multi-labeling
VAM: Variance adjusted Mahalanobis
CDF: Cumulative distribution function
TME: Tumor microenvironments
ChIMP: CAMML with the integration of marker proteins
